# Evaluation of Anatomic Variations in Maxillary Sinus with the Aid of Cone Beam Computed Tomography (CBCT) in a Population in South of Iran

**Published:** 2016-03

**Authors:** Shoaleh Shahidi, Barbad Zamiri, Shahla Momeni Danaei, Setareh Salehi, Shahram Hamedani

**Affiliations:** 1 Biomaterials Research Center, Dept. of Oral and Maxillofacial Radiology, School of Dentistry, Shiraz University of Medical Sciences, Shiraz, Iran.; 2 Dept. of Oral and Maxillofacial Surgery, School of Dentistry, Shiraz University of Medical Sciences, Shiraz, Iran.; 3 Orthodontics Research Center, Dept. of Orthodontics, School of Dentistry, Shiraz University of Medical Sciences, Shiraz, Iran.; 4 Undergraduate Student, Student Research Committee, School of Dentistry, Shiraz University of Medical Sciences, Shiraz, Iran.; 5 Dental Research Development Center, School of Dentistry, Shiraz University of Medical Sciences, Private Practice, Shiraz, Iran.

**Keywords:** Cone-beam Computed, Tomography, Normal Variations, Maxillary Sinus, Antrum, Pneumatization

## Abstract

**Statement of the Problem:**

Anatomic variations of the maxillary sinus can be detected in cone-beam computed tomography (CBCT) and may assist to locate the posterior superior alveolar artery (PSAA) and define the maxillary sinus morphology more accurately for a more strict surgical treatment plan.

**Purpose:**

The study aimed to determine normal variations of the maxillary sinus with the aid of CBCT in a sample population in south of Iran.

**Materials and Method:**

This cross-sectional prevalence study was based on evaluation of 198 projection data of CBCT scans of some Iranian patients aged 18-45 who referred to a private oral and maxillofacial radiology center in Shiraz from 2011 to 2013. CBCT scans were taken and analyzed with NewTom VGi device and software. The anatomic variations which were evaluated in the axial images included the presence of alveolar pneumatization, anterior pneumatization, exostosis, and hypoplasia. Moreover the location and height of sinus septa, and the location of PSAA were assessed. SPSS software (version 17.0) was used to analyze the data.

**Results:**

In a total of 396 examined sinuses, maxillary sinus alveolar pneumatization was the most common anatomic variation detected. Anterior pneumatization was detected in 96 sinuses (24.2%). Antral septa were found in 180 sinuses (45.4%) and were mostly located in the anterior region. Meanwhile, PSAA was mostly detected intra-osseous in 242 sinuses (65.7%).

**Conclusion:**

Anatomic variations of the maxillary sinus were common findings in CBCT of the maxilla. Preoperative imaging with CBCT seems to be very helpful for assessing the location of PSAA and the maxillary sinus morphology, which may be used to adjust the surgical treatment plan to yield more successful treatments.

## Introduction

The maxillary sinus in adults is composed of a pyramid-shaped cavity in the facial skull with its base at the lateral nasal wall and its apex extending up to the zygomatic process of the maxilla.[1] It can exhibit anatomic variations such as pneumatization, hypoplasia, antral septa, exostosis, and variations in location of the arteries.[2] All the surgical interventions in the posterior maxillary region require detailed knowledge of the maxillary sinus anatomy and possible anatomical variations.[3]

Maxillary sinus hypoplasia (MSH) is the under development of the maxillary sinus, which can occur during embryological development or later in life due to trauma, iatrogenic, or structural causes.[4] The narrow infundibular passage associated with the absence of a natural ostium would result in mucosal thickening of the hypoplastic sinus.[5] Furthermore, MSH causes the proximal extension of the lateral nasal wall and subsequently makes the surgical procedures difficult.[2, 6]

Maxillary sinus septa are barriers of cortical bone. The shape is described as an inverted gothic arch arising from the inferior or lateral walls of the sinus that divide the maxillary sinus floor into multiple compartments, known as recesses.[1, 3] These septa were first analyzed by Arthur S. Underwood, an anatomist who reported their prevalence and characteristics and these septa were afterwards, referred to as Underwood’s septa.[1]

In a systematic review published by Pommer *et al.*,[7] electronic and hand searching of English literature were employed to identify the studies published from 1995 to 2011.They reported that the observed septa were at least 2-4 mm in height, and 7.5 mm on average. They were present in 28.4% of 8923 investigated sinuses (95% confidence interval: 24.3–32.5%). Septa were located in premolar, molar, and retromolar regions in 24.4%, 54.6% and 21.0% of cases, respectively. Their orientation was transverse in 87.6%, sagittal in 11.1%, and horizontal in 1.3% of the studied cases. Complete septa (dividing the sinus into two separate cavities) were found only in 0.3% of samples. Other rare conditions included multiple septa in one sinus (4.2%) and bilateral septa (17.2%). Moreover, the diagnosis of septa by using panoramic radiographs yielded incorrect results in 29% of cases.[7]

Septum removal before sinus augmentation is a preferred procedure, as with the septum in place, there is a high possibility of membrane perforation that results in maxillary sinusitis.[3] Dental panoramic radiography, computed tomography (CT), and cone beam computed tomography (CBCT) have all been used to identify the maxillary sinus septa.[8-15] CBCT is a technique that has been proposed for maxillofacial imaging during the last decade and was first reported by Mozzo *et al.*[1, 16]

The posterior superior alveolar artery (PSAA) and infraorbital artery (IOA) are the branches of maxillary artery that supply the lateral sinus wall and the overlying membrane. The blood supply of the maxillary sinus and Schneiderian membrane comes from the maxillary artery.[17] The presence of this artery was first mentioned by Strong in 1934.[18]

The branches of maxillary artery should be taken into consideration because of the potential risk of bleeding during the procedures such as open sinus lift surgery, horizontal osteotomy of the maxilla, Le Fort I fracture treatment, and Caldwell-Luc surgeries.[17-20]

In a study done by Rahpeyma *et al.*, thirty five CBCT scans from 35 dentate patients were selected in coronal sections of the second premolar (P2), first molar (M1), and second molar (M2). The presence of alveolar antral artery in each situation was determined and the bone thickness in the region of alveolar antral artery was measured perpendicular to the lateral wall of the maxilla. The alveolar antral artery was present in 67.1% of CBCTs.[19]

Many imaging techniques such as panoramic, waters, Caldwell, CT, MRI, and CBCT can be used to study the maxillary sinuses region. For a long period, skull projections including Waters, Caldwell and lateral sinus were used for evaluation of the paranasal sinuses. Waters view is useful for gross evaluation of the maxillary sinus especially for localized mucosal thickening along the sinus floor, generalized thickening of the mucosal lining around the entire wall of the sinus, and near-complete or complete radiopacification of the sinus. Plain films are no longer considered to be a part of the primary imaging modalities. At best, they give only an overview of the anatomy and underlying pathoses, as they are limited to display three-dimensional (3D) structures in a two-dimensional (2D) plane. CT and MR imaging have the advantage of being able to show fine anatomic details in serial topographic sections, and thus excluding the gross volume averaging which is a characteristic feature in plain films. In fact, in most cases, when a plain-film study shows the probable presence of the disease, a CT or MR imaging is consequently obtained.[21-22]

CBCT uses a cone- or pyramidal-shaped beam to acquire multiple projections in only one rotation. On the other hand, multislice computed tomography (MSCT) employs fan-shaped beams rotating around the patient to acquire multiple image slices.[2, 23-24]

CBCT may be recommended as a low-cost dose-sparing technique compared with standard medical computed tomography scans (MDCT), though CBCT has slightly more radiation exposure than routine panoramic radiography for dentomaxillofacial imaging.[1, 16, 25-31]

The effective dose from a standard dental protocol scan with MDCT is 1.5 to 12.3 times greater than comparable medium–field of view dental CBCT scans according to International Commission on Radiological Protection (ICRP 2007).[16] Moreover, beam-hardening artifacts due to dental materials (like amalgam and crowns) and implants are weaker at CBCT than at MSCT.[32]

To minimize the risk of postoperative complications of maxillary sinus floor lift and other surgeries in this region, it is crucial to be familiar with different anatomic and pathologic findings in sinus.[1, 8-13,29, 33] As the maxillary sinuses are significant anatomic structures in dental practice that their exact and definitive radiological assessment is necessary, and considering CBCT as an important diagnostic image modality in dentistry, the recognition of anatomic variations of the maxillary sinuses in CBCT is noteworthy.[1-2]

Several studies have been performed on the prevalence of anatomic variations in different populations; however, our information is insufficient regarding the Iranian population. Therefore, the aim of the present study was to determine the maxillary sinus normal variations with the aid of CBCT in a sample of population resident in south of Iran. 

## Materials and Method

This cross-sectional prevalence study was based on evaluation of CBCT scans of some Iranian patients aged 18-45, who referred to a private oral and maxillofacial radiology center in Shiraz, from 2011 to 2013. To this end, 198 CBCT images of originally Iranian patients were selected from the archive of adults who needed those images for other justified reasons.

All CBCT images of the adult patients which showed maxillary sinuses were included in the study sample. The CBCT images of patients with systemic problems and evidence of previous trauma or manipulation of the maxillary sinuses, as well as those images with any sign of pathologic changes in maxillary sinuses were excluded from the study.

CBCT scans were taken with NewTom VGi device (covering the maxillary region, focal spot=0.3 or 0.15mm, scanning time=90s). They were analyzed by the related NewTom software on a multiplanar reconstruction window in which the axial, coronal, and sagittal planes could be visualized in 0.3 mm intervals. 

To standardize the reading and interpreting of the CBCT images, two researchers were trained and calibrated by using 10% of the samples in a one-week pilot study before the data collection began.

The anatomic variations evaluated in the axial images were alveolar pneumatization, anterior pneumatization, location and height of sinus septa, exostosis, hypoplasia, and location of the PSAA.

The septa height more than 2 mm (the important factor in sinus floor elevation) was registered and location of the septa was divided into 3 groups of anterior, middle, and posterior. The distances from the artery to the medial sinus wall were determined and the locations of the artery were categorized as intra-osseous (A), below the membrane (B), and on the outer cortex of the lateral sinus wall(C). In the presence of two alveolar antral arteries in a coronal section, the larger one was considered. The presence of septa was evaluated in the coronal and sagittal images.

The SPSS software (Ver. 17.0) was used to analyze the data. The descriptive analysis was presented as frequency, mean±SD, 95% confidence intervals (CI), and the range. 

## Results

In a total of 198 CBCT images, 396 sinuses were evaluated in which 130 cases belonged to females (65.7%) and 68 to males (34.3%).

Maxillary sinus alveolar pneumatization (maxillary sinus extension into alveolar process) was the most common anatomic variation detected, observed in 228 sinuses (57.5%). The pneumatization sites were multiple in 90 (65.2%) and single in 48 cases (34.8%). (Table 1)

The anterior pneumatization was detected in 96 sinuses (24.2%), 40 single (58.8%) and 28 multiple (41.2%). Scalloped margin between teeth roots was observed in 100 sinuses (25.2%). 

Maxillary sinus hypoplasia was detected only in 26 sinuses (6.5 %) that included 14 unilateral (70%) and 6 bilateral (30%) cases. (Table 1)

**Table 1:** Frequency of normal variations

		**Hypoplasia**	**Exostosis**	**Scalloped Margin**	**Septa**	**Alveolar** **Pneumatization**	**Anterior** **Pneumatization**
All sinuses		26 (6.5%)	13 (3.2%)	100 (52.2%)	180 (45.4%)	228 (57.5%)	96 (24.2%)
Patients	Unilateral	14 (70%)	3 (37.5)	44 (61.1%)	70 (56%)	48 (34.8%)	40 (58.8%)
	Bilateral	6 (30%)	5 (62.5)	28 (38.9%)	55 (44%)	90 (65.2%)	28 (41.2%)
	Total	20 (100%)	8 (100%)	72(100%)	125 (100%)	138 (100%)	68 (100%)

Exostosis was identified in 13 sinuses (3.28%). Antral septa were found in 180 sinuses (45.4%); bilateral in 55 (44%) and unilateral in 70 cases (56%). (Table 1)

**Figure 1:**
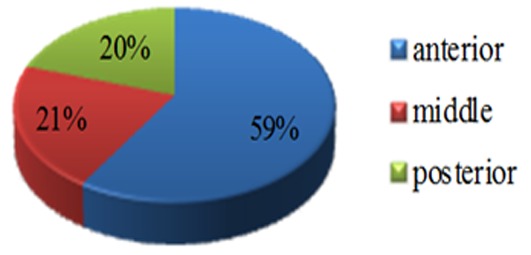
Locations of septa

Sinus septum was in anterior region in 106 (58.9%), middle in 38 (21.1%), and posterior in 36 (20%) of sinuses containing septa. (Figure 1) Sixty four (35.5%) of the septa were also detected in coronal slices and 112 (62.2%) were viewed in sagittal sections, as well. Ninety eight (54.4%) of all septa divided the sinus into 2 cells and 8 (4.4%) into 3 separate cells.

The minimum and maximum height of the right sinus septum was measured to be respectively 2.1 and 23 with the mean±SD=8.17±3.6. These numbers for the left sinus septum were 3 and 25.6, respectively, with the mean±SD=8.28±4.29. (Table 2)

**Table 2:** Measurements of sinus size

	**Anterior-posterior**				**Medial-lateral**			
	**Min**	**Max**	**Mean**	**Std**	**Min**	**Max**	**Mean**	**Std**
Right	26.5	48.6	37.2	4.0	5.4	20.8	15.0	3.1
Left	25.8	45.9	37.2	3.8	7.5	22	14.7	3.1

**Figure 2:**
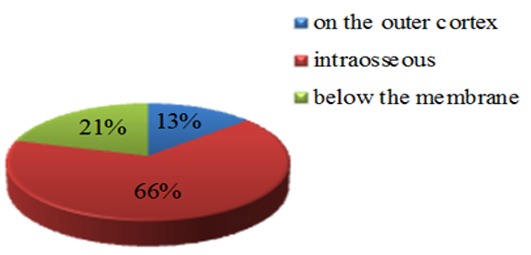
Location of posterior superior alveolar artery (PSAA)

PSAA was absent in 28 sinuses (7%). Figure 2 shows the percentage of different locations of the artery in those images in which artery was detected. The artery was located on the outer cortex of the sinus wall in 50 cases (13.5%). Moreover, the artery was intra-osseous in 242 sinuses (65.7%), and below the membrane in 76 sinuses (20.6%). The minimum and maximum distance from the artery to the medial wall on the right sinus was 10.60 and 37.50, respectively (mean±SD=24.8657± 4.94112), and 13.20 and 36.60 on the left side (mean± SD=24.8214±4.71998).

## Discussion

Preoperative imaging is very important and clinically relevant for the detection of maxillary sinus variations and pathologic problems. In 3D imaging, the treatment plan can be modified and the outcome of pre-prosthetic surgery in posterior maxilla can become more predictable.[29]

The alveolar antral artery is an important anatomical structure within the lateral maxillary sinus wall. The presence of this artery was first mentioned in an article by Strong published in 1934.[18]

According to Ilgüy *et al.*[17] and Khajehahmadi *et al.*,[34] an important point especially in the Caldwell-Luc and open sinus lift surgery is that the maxillary sinus wall has a considerable vascular anastomosis. Damage to the vessels of the bone can cause bleeding, may obscure the physician’s line of sight, and may lead to perforation of the Schneiderian membrane, all of which prolong the operation and assessment of the sinus membrane reflection.[17, 34]

In the present study, the presence and location of the PSAA was observable through CBCT scans. The artery was observed in 93% of the sinuses and was mostly intraosseous (65.7%). The success rate for identifying the artery was higher than that reported by Ilgüy *et al.* (89.3%),[17] Güncü *et al.* (64.5%),[35] Elian *et al.* (52.9%),[36] Mardinger *et al.* (55%),[37]and Kim *et al.* (52%).[8] This may be related to the method and resolution of the advanced images that were used to detect and describe the artery.

CBCT provides accurate and reliable linear measurements for reconstruction and imaging of dental and maxillofacial structures. In the study by Ilgüy *et al.* in 2013, the distance of the artery from the medial sinus wall was 13.92±2.84 mm;[17] while in our study, this number was 24.8657±4.94mm on the right sinus and 24.8214±4.71mm on the left sinus. These differences may be explained by the anatomic variation in the positions of arteries and the populations that were examined.

According to Naitoh *et al.*, antral septa was defined as a pointed bone structure and maxillary sinus exostosis as a rounded bone structure, both of which originated from any maxillary sinus wall.[12] Van den Bergh *et al.* emphasized that antral septa, detected in almost half of the CBCT exams, might increase the risk of sinus membrane perforation during the maxillary sinus floor elevation surgery.[39] Abrahams *et al.* and Aimetti *et al.* reported that the accidental perforation of this membrane could lead to development of acute or chronic sinusitis, and subsequent bone graft resorption.[40-41] Furthermore, antral septa should be considered in lifting the bone plate and sinus membrane during surgery.[42]

With normal sinus anatomy, preparation and horizontal rotation of a trap door in the maxillary sinus wall is a common procedure and is possible when the Schneiderian membrane is sufficiently lifted. The most frequent complication in this procedure would be the tearing of the sinus membrane, which is in turn correlated to the presence of septa in the maxillary sinus.[39, 42-43]

The presence of maxillary sinus septa can be detected in panoramic radiographs. However, CT and CBCT are definitely the preferred imaging techniques for the assessment of this anatomic variation. Krennmair *et al.* found that panoramic radiograph can lead to false diagnosis regarding the positive or negative identification of septa in 21.3% of cases. They stated that CT scanning was the preferred imaging method for detecting the presence (or absence) of sinus septa since it allows the high-resolution imaging of delicate bony structures.[10]

According to Pommer *et al.*,[7] diagnosis of sinus septa by using 2D panoramic radiographs compared with 3D computed tomography produced incorrect results in 29% of cases. They claimed that panoramic radiographs may not image those sinus septa with sagittal orientation and might, thus, lead to false assumption of narrow internal sinus anatomy and subsequent non-augmentation of the medial portion of the sinus cavity. The pre-operative radiographic imaging of sinuses should be made concerning the surgical complications and following modifications that can be possibly made to avoid these consequences.[7] In case of sinus floor augmentation; it ranges from modification in the surgical access strategy (or window design) to change in implant positions or even complete avoidance of bone graft surgery. Considering the high prevalence and significant morphologic variability in sinus septa in the above-mentioned investigation, 3D radiological imaging prior to sinus floor augmentation surgery may help to reduce complications in the presence of maxillary sinus septa.[7]

The range of septa prevalence was found to be 24-33% in a review article (four studies) published by Katranji *et al.*[44] and 13-35% in a review of 11 studies performed by Maestre-Ferrín *et al.*[45] which included investigations that used panoramic radiographs. The prevalence of sinus septa was found to be 16.1% in Güncü *et al.*’s study,[35] 16% in Krennmair *et al.*’s study,[10-11] and 26.5% in Kim *et al.*’s study when CT was used to assess the sinuses.[8]

In a study performed in Iran with spiral CT-scan, the prevalence of at least one septum was 29.5%.[3] The results of the present study revealed that sinus septa were observed in 45.4% of the 396 sinuses. The results of the CT evaluation of the maxillary sinus septa in the reported articles are not consistent with those of the present study. On the other hand, much higher percentages have been reported with CBCT, and they are close to the results obtained by the current study.

The prevalence of sinus septa was reported to be 55.2% by Ilgüy *et al.*,[17] 58% as found by Orhan *et al.*,[1]and 47% as reported by Neugebauer *et al.*[46] In another study, Lana *et al.* stated that the prevalence of antral septa was 44.4%.[2] These differences could be attributed to the different imaging modalities employed in these studies.

Complete septa (dividing the sinus into two separate cavities) were found only in 0.3% as reported in the systemic review done by Pommer *et al.*,[7] and in 25.2 % (n=100) of the sinuses in our study.

The analysis of the position of septa showed that sinus septum was in anterior region in 106 (58.9%), middle in 38 (21.1%), and posterior in 36 (20%) of sinuses containing septa.

In the study carried out by Faramarzie *et al.* in Iran, most of the septa (53.84%) were reported to be in the middle region.[3] In some studies, a greater number of incidence was found in the middle regions;[1, 10-11,47]while, several other studies detected them in the anterior [9, 13] or posterior regions.[1, 9, 13] Selcuk *et al.* found that the distribution of septa in the anterior region was higher than in the posterior region (20.3% and 2.5%, respectively).[48] Hadchiti *et al.* reported no statistically significant difference in the antero-posterior location; that is, 55 septa were posteriorly located in the molar region (28.65%), 75 were near the first and second premolar (middle area) (39.06%), and 67 septa were detected in the anterior area (32.29%).[49] According to Faramarzie *et al.*’s study, the sequence of tooth extraction can also affect the formation of antral septa in different regions of the sinus.[3]

In our measurements, the mean height of septa was 8.22mm; while, previous studies reported different heights for the septa ranging from 5.6 to 20.6 mm.[1, 9, 11-13,46-47]

Alveolar pneumatization was reported in approximately 50% of the population in the study by Schuh *et al.*,[50] 100% of the patients in Lana *et al.*’s research,[2] and was present in 228 sinuses (57.5%) in our study. Gosau *et al.* stated that atrophy of the maxilla caused by edentulism was characterized by vertical and horizontal bone loss.[51] The maxillary sinus pneumatization, particularly the alveolar extension, can intensify the problem of reminiscent bone caused by atrophy of the maxilla, leaving only few millimeters of bone for implant insertion.[2, 52]

The frequency of maxillary sinus hypoplasia was reported to be 4% in Shiki *et al.*’s study,[53] 4.8% in Lana *et al.*’s research,[2] and 6.5% in the current investigation. Shiki *et al.*,[53] found the antral exostosis in 3% of the population, Lana *et al.*[2] reported it to be 2.6%, and it was 3.2% in our study. These differences may be due to different sample sizes, the resolution of CBCT units which were used, as well as the anatomic variations in different populations.

Fernandes reported that the size of maxillary sinus differed among various ethnics in different populations.[54] They experienced that 48.6% of European maxillary sinuses had larger maxillary sinus volumes than Zulu sinuses. Moreover, Butaric *et al.* reported that the Peruvian samples had lower antral volume than the Australian samples.[55] Another study reported that the mean maxillary sinus volume in girls was larger than that in boys aged 4-9 in a Japanese population.[56] Therefore, the current study seems to be justified concerning these differences observed in different ethnics. Investigating the prevalence of these important anatomical features in Iranian population, especially in different parts of the country would be helpful for young surgeons in this population.

## Conclusion

The anatomic variations of maxillary sinus are common findings in CBCT of the maxilla. Since some of these conditions can modify the surgery planning to more specialized procedures, they are crucial to be recognized in dental practice. Inevitably, preoperative imaging with CBCT is helpful for assessing the location of the PSAA, maxillary sinus morphology, and normal variations which may be used to adjust the surgical treatment plan to yield more successful treatments. 
